# The effect of heightened awareness of observation on consumption of a multi-item laboratory test meal in females

**DOI:** 10.1016/j.physbeh.2016.04.044

**Published:** 2016-09-01

**Authors:** Eric Robinson, Michael Proctor, Melissa Oldham, Una Masic

**Affiliations:** University of Liverpool, UK

**Keywords:** Demand characteristics, Experimenter effects, Laboratory methods, Eating behaviour, Awareness, Observer effect, Energy intake

## Abstract

Human eating behaviour is often studied in the laboratory, but whether the extent to which a participant believes that their food intake is being measured influences consumption of different meal items is unclear. Our main objective was to examine whether heightened awareness of observation of food intake affects consumption of different food items during a lunchtime meal. One hundred and fourteen female participants were randomly assigned to an experimental condition designed to heighten participant awareness of observation or a condition in which awareness of observation was lower, before consuming an *ad libitum* multi-item lunchtime meal in a single session study. Under conditions of heightened awareness, participants tended to eat less of an energy dense snack food (cookies) in comparison to the less aware condition. Consumption of other meal items and total energy intake were similar in the heightened awareness vs. less aware condition. Exploratory secondary analyses suggested that the effect heightened awareness had on reduced cookie consumption was dependent on weight status, as well as trait measures of dietary restraint and disinhibition, whereby only participants with overweight/obesity, high disinhibition or low restraint reduced their cookie consumption. Heightened awareness of observation may cause females to reduce their consumption of an energy dense snack food during a test meal in the laboratory and this effect may be moderated by participant individual differences.

## Highlights

• We examined the effect of heightened awareness of observation on food intake.
• This caused female participants to eat less of an energy dense snack food.
• This effect was moderated by weight status and trait eating behaviour measures.
• Heightened awareness of observation had little effect on intake of other foods.

## 1 Introduction

Human eating behaviour is often studied in the laboratory [1], [2], [3]. A potential methodological issue of such research is social desirability on the part of the participant [4]. Because people form impressions and stereotypes based on how much or what a person eats [5], [6], [7], [8], if a participant were to believe that their food consumption was being measured in a study, this may cause them to alter their eating behaviour [4], [9]. In line with this notion, a recent meta-analysis has shown that when females feel as though their behaviour is being observed in the laboratory they significantly reduce their energy intake [10]. However, a limitation of this meta-analysis was that most studies had only measured intake of energy dense snack foods, such as cookies. There is reason to believe that consumption of other food types may be affected by heightened awareness of observation. For example, when people self-report their food intake a wide variety of different food types are under-reported, including snack foods and meal foods high in fat [11], [12]. Under-reporting of food intake is likely to be in part be caused by self-presentation concerns [13]. Likewise, in a recent study, Stubbs et al. [14] found that, when female participants were led to believe that their food intake was being closely measured, they reduced their energy intake and this was apparent for energy derived from fat, protein and carbohydrates. However, in a different study, Thomas et al. [15] told female participants that there was a hidden set of scales weighing their plate during a meal and found little evidence that this experimental manipulation influenced consumption of two different test foods (pasta followed by cookies).

Given that a large number of laboratory eating behaviour studies involve consumption of multi-item test meals [3], [16], [17], [18], [19], in the present study our aim was to examine whether heightened awareness of observation affects consumption of different food items (e.g. savoury and sweet, high and low fat) during a lunchtime test meal. This was to assess whether awareness of observation may influence intake of the high fat ‘unhealthy’ foods offered differently to the low fat foods, on account of self-presentation concerns. A novel secondary aim of the present work was to explore whether individual differences moderated the influence that heightened awareness of observation has on food intake. In the aforementioned recent meta-analysis, whether participant weight status or eating traits [20] (e.g. restrained, disinhibited or emotional eating), moderated the influence of heightened awareness of observation on food intake was not examined due to a lack of data. However, there has been some suggestion that overweight individuals are particularly self-conscious of their weight and eating behaviour [21], [22], so could be more likely to eat minimally when awareness of observation is heightened. Likewise, it has been suggested that restrained eaters are particularly conscious of how their eating behaviour is perceived by others [10], [23], as indicated by dietary restraint predicting greater under-reporting of energy intake [24]. Moreover, disinhibition and emotional eating have been shown to predict food intake in the laboratory [25], [26], [27] and it is conceivable that they may be moderators of the influence that heightened awareness of observation has on food consumption.

We hypothesised that heightened awareness of observation would affect participant food intake. More specifically, in line with recent work from our laboratory [4], we predicted that heightened awareness would reduce consumption of cookies (a high fat, ‘unhealthy’ food item) and we tentatively hypothesised that the consumption of other high fat meal items may also be reduced. With regard to individual differences, we hypothesised that heightened awareness may reduce food intake among participants who would be likely to have raised self-presentation concerns, e.g. overweight and obese participants and restrained eaters. We did not have strong a-priori hypotheses concerning the moderating effects trait disinhibition or emotional eating may have on the influence of heightened awareness.

## 2 Method

### 2.1 Sample

To be consistent with existing research on heightened awareness of observation of food intake [10] and to be able to recruit a homogenous sample of participants, we recruited females only. We powered the study based on a comparable study that had been recently conducted in our laboratory [4]. In that study, heightened awareness of observation had a statistically large effect on food consumption. A power calculation with 95% power, *d* = 0.8, *p* < 0.05 indicated that we would require 84 participants to detect significant differences in food intake between the two conditions in the present study. However, we were mindful that the size of any meaningful effects observed in the present study (e.g. secondary moderation analyses) may be smaller in size. Thus, because of practical constraints we made a pragmatic a-priori decision to recruit a minimum of 84 participants, but to collect data for up to nine months from study start and recruit as many eligible participants as was feasible in this time frame.

### 2.2 Participants

One hundred and twenty two participants were recruited from 1st year psychology students at a UK university campus, university staff or the surrounding community. To disguise the aims of the research, the study was advertised as examining ‘mood, cognition and satiety’. Participants were reimbursed a small cash sum or could instead receive course credit (1st year psychology students only). Participants who registered interest in the study were only eligible for participation if they were female, aged 18 years or older, had no known history of food allergies and were not vegetarians. All procedures were approved by the University of Liverpool Research Ethics Committee. Written informed consent was obtained from all participants.

### 2.3 Cover story

Participants were led to believe that the study assessed the influence of meal consumption on mood and cognitive ability. To corroborate the cover story, participants were asked to complete mood measures before and after being served the lunch meal. Shortly after the meal participants were also asked to complete a short cognitive task which involved line tracing the outline of two shapes as accurately as possible using their non-dominant hand.

### 2.4 Design

Participants were randomly assigned to one of the two conditions in a between-subjects design; ‘heightened awareness’ or ‘less aware’. Either a male or female researcher aged between 18 and 24 years old oversaw laboratory sessions (5 researchers in total ran individual study sessions). We examined whether the researcher running the session, or their gender, affected any of our reported results (when controlled for and when included as a factor in analyses) and found no evidence that they did, so did not include this as a factor in our main analyses (see online supplemental materials).

### 2.5 Manipulation

In the ‘heightened awareness’ condition, prior to consuming their lunchtime meal participants were given a set of written instructions which stated; ‘*In this study we want to examine your eating behaviour and food intake* (*i.e. how much you eat of each food*). *Because of this*, *in this section of the study you will be served some lunch foods*. *You can eat as little or as much of each food as you like and after you have finished eating we will later weigh each plate to work out how much you have eaten*. *Also*, *after you have finished eating you will be asked to complete a simple pen and paper cognitive task*.’ In the ‘less aware’ condition the instructions read; ‘*In this study we want to examine cognitive performance and mood at different times of the day* (e.g. *mid-morning*, *after eating lunch*, *in the evening* etc.). *Because of this*, *in this section of the study you will be served some lunch foods*. *You can eat as little or as much of each food as you like*. *After you have finished eating you will be asked to complete a simple pen and paper cognitive task*.’ In both conditions the researcher verbally checked that the participant understood the instructions.

### 2.6 Test foods

Participants were served a multi-item lunch meal on a tray which consisted of commonly consumed UK lunch items on well stocked individual plates (see Table 1) and a large glass of water. We selected the lunch meal food items in order to have a balance of food items which were high and low in fat and which most participants would perceived as being ‘healthy’ and ‘less healthy’. Data collected during the session supported this with participants rating the high fat options (cookies, crisps and sausage rolls) as more unhealthy and the low fat options (sandwich, rice cakes and grapes) as more healthy on 100 mm VAS scales (see procedure).

### 2.7 Procedure

Participants attended a session that took place between 12:00 pm–2.30 pm and were asked to abstain from eating in the two hours prior to the session. Sessions took place in a cubicle in the Kissileff eating behaviour laboratory. On arrival the researcher described the cover story to the participant and explained what would happen during the session. After providing consent, participants completed a medical history questionnaire to ensure that they did not have any food-related allergies. Participants next provided demographic information (gender, age, ethnicity) and completed a set of 11 paper based 100 mm visual analogue scales (left hand anchor: not at all, right hand anchor: extremely) to measure appetite (hunger, fullness, desire to eat; e.g. ‘how hungry do you feel right now?’) and various mood dimensions in line with the cover story. At the end of the mood ratings, the experimental condition manipulation information was provided. When participants had read the instructions they were asked to alert the researcher (using a buzzer). In order to ensure that participants had read and understood the information provided the researcher then asked participants to confirm that they understood the aims and verbally reiterated the instructions. The researcher then brought the lunch meal to the participant and explained that they had up to 20 min to eat as much or as little as they desired. At this point the researcher left the room and timed meal duration. When the participant had finished eating or after the timed 20 min had elapsed (this occurred in 15% of participants), the researcher returned and removed the lunch.

After lunch, to further corroborate the cover story participants were asked to complete the line tracing task. They then repeated the appetite and mood VAS. Participants were then asked to rate the palatability of the meal items (including liking of each individual item in the meal and how much they would normally like eating each meal item) to determine acceptability, before rating how ‘unhealthy’ they believed each item was using 100 mm VAS scales (anchors: not at all, extremely). After this participants were asked to write down what they thought the aims of the study were, before completing a short questionnaire about their experience in the study. Embedded within this questionnaire was a manipulation check item ‘I felt as though the amount of food I was eating would be measured by the researcher’ (5 point Likert scale, strongly disagree to strongly agree). Participants then completed the 21 item Three Factor Eating Questionnaire [20]; this resulted in sub-scores [1], [2], [3], [4] for dietary restraint, disinhibition and emotional eating. Next participants were weighed and measured in order to calculate BMI. Finally, participants were debriefed, thanked and compensated for their time.

### 2.8 Main analysis strategy

We first examined whether there were any between condition differences for participant characteristics and baseline variables; age, baseline hunger, BMI and eating trait sub-scores (restraint, disinhibition, emotional eating) of the TFEQ. Our primary research question was whether consumption of meal items was significantly reduced in the heightened awareness condition compared to the less aware condition. To test this we used independent sample *t*-tests for each of the test meal items. A secondary aim was to examine potential moderators of the effect that heightened awareness had on food consumption. To test this we examined whether participant weight status (normal weight vs. overweight/obese) or any of the TFEQ sub-scores interacted with the effect of condition in a series of 2 × 2 between-subjects ANCOVAs; we categorised participants as high or low scorers for each of the TFEQ sub-scales using median splits [28], [29]. When examining the independent moderating effect of participant BMI we controlled for the three TFEQ sub-scales as covariates in the ANCOVA. Likewise, when examining the independent moderating effect of a TFEQ sub-scale grouping we controlled for participant BMI and the remaining two TFEQ sub-scales. If we observed evidence of moderation, we then conducted separate independent sample *t*-tests and because these analyses were exploratory in nature, we did not correct the statistical significance level of these tests for multiple comparisons. See online supplemental materials for correlations between BMI and TFEQ sub-score variables.

## 3 Results

### 3.1 Sample characteristics

Of the 122 recruited participants, eight participants were excluded from analyses resulting in a final sample of 114. Three of the participants were excluded as they did not follow study instructions (e.g. they did not complete the questionnaire measures provided), and five participants directly guessed the aims of the study (e.g. they wrote that they believed the study was about how being observed affected the amount they ate). The mean age of the final sample was 24.3 years (SD = 10.5) and most participants were of Caucasian descent (87%). Measurement error resulted in BMI data being unavailable for three participants and was therefore classified as missing for these participants. The mean BMI of the sample (*N* = 111) was 23.6 kg/m^2^ (SD = 4.60).

### 3.2 Cover story

As noted, only five of all recruited participants (4%) guessed the study aims (one from the less aware condition and four from the heightened awareness condition). The majority of the remaining participants believed the study was about the influence that eating had on mood and/or their ability to complete the line tracing task, indicating that the cover story was effective.

### 3.3 Condition differences for participant characteristics

The two conditions did not significantly differ from each other on BMI, age, baseline hunger or any of the three TFEQ sub-scale scores (ps > 0.10). See Table 2.

### 3.4 Consumption of test-meal items

#### 3.4.1 Cookie intake

Participants in the heightened awareness condition tended to consume fewer grams of cookies 26% less than participants in the less aware condition, although this difference (*p* = 0.05, *d* = 0.37) was not statistically significant at *p* < 0.05. See Table 3.

#### 3.4.2 Other meal items intake and total energy intake

Participants in both conditions consumed a similar amount of sandwiches, sausage rolls, crisps, rice cakes and grapes, as well as having a similar total lunchtime energy intake (all ps > 0.25). See Table 3.

### 3.5 Awareness of observation

#### 3.5.1 Manipulation check

Participants in the heightened awareness condition (M = 4.33, SD = 0.70, 1–5 Likert scale) scored significantly higher than participants in the less aware condition (M = 3.65, SD = 1.04) on the manipulation check measure (t (112) = 4.07, *p* < 0.001, *d* = 0.76), indicating that the heightened awareness manipulation did cause participants to believe more strongly that their food intake was being measured. In the heightened awareness condition, 91% (49/54) of participants either strongly agreed or agreed that they felt as though their food consumption would be measured, whilst in the less aware condition this was 67% (40/60) of participants. In line with the between condition differences for food intake, participant awareness of observation was significantly correlated with cookie consumption (*r* = − 0.22, *p* = 0.016), but not with consumption of any other food items (ps > 0.05).

#### 3.5.2 Sensitivity analyses

We conducted a further analysis only including participants from the heightened awareness condition who reported that they believe their food intake was being measured (*n* = 49) and participants from the less aware condition who did not report that they believed their food intake was being measured (*n* = 20). As such, the difference between these two conditions on the manipulation check was now larger (heightened awareness: M = 4.49, SD = 0.51, less aware: M = 2.35, SD = 0.49). Moreover, cookie consumption was now clearly significantly lower (t (67) = 3.25, *p* = 0.002, *d* = 0. 74) in the heightened awareness condition (M grams = 17.14, SD = 13.90) compared to the less aware condition (M grams = 30.76, SD = 19.77). In a further sensitivity analysis of the awareness data we examined the effect of just removing the 5 participants from the heightened awareness condition who did not report that they believed their food consumption was being measured. The marginal effect in the full sample (*p* = 0.05, *d* = 0.37) of participants eating fewer cookies in the heightened awareness condition vs. less aware condition remained in the same direction and was now statistically significant with these 5 participants removed (*p* = 0.015, *d* = 0.48). In both of these sensitivity analyses the results of the reported non-significant differences between the two conditions for consumption of the other meal items remained the same (ps > 0.05).

### 3.6 Moderation analyses

Because we only found evidence that cookie intake was affected by condition, our main moderation analysis examined cookie intake. Note: for full moderation results (i.e. full results of main effects and covariates from ANCOVAs) see online supplemental materials.

#### 3.6.1 BMI

Because there were only a small number of underweight (*N* = 8) participants (BMI < 18.5) we excluded these participants from the analysis, resulting in two between subjects groups; normal weight participants (BMI = 18.5–24.9) and overweight/obese participants (BMI ≥ 25). In the ANCOVA there was a significant interaction between condition and weight status (F (1, 96) = 4.91, *p* = 0.03, ηp^2^ = 0.05). To interpret the interaction we examined the effect of condition among normal weight participants and overweight/obese participants separately using independent samples *t*-tests. See Table 4. Overweight/obese participants consumed significantly fewer grams of cookies in the heightened awareness condition vs. the less aware condition. Cookie intake did not differ between conditions among the normal weight participants.

#### 3.6.2 Restraint

In the ANCOVA there was a significant interaction between condition and restraint (F (1, 104) = 4.39, *p* = 0.04, ηp^2^ = 0.04). Unrestrained participants consumed significantly fewer grams of cookies in the heightened awareness condition vs. the less aware condition. Cookie intake did not differ between conditions among the highly restrained participants. See Table 4.

#### 3.6.3 Disinhibition

The interaction between condition and disinhibition approached statistical significance (F (1, 104) = 3.80 *p* = 0.05, ηp^2^ = 0.04). Highly disinhibited participants consumed significantly fewer grams of cookies in the heightened awareness condition vs. the less aware condition (*t*-test results). Cookie intake did not differ between conditions among the low disinhibition participants. See Table 4.

#### 3.6.4 Emotional eating

There was no significant interaction between condition and emotional eating (F (1, 104) = 0.77, *p* = 0.38, ηp^2^ < 0.01), indicating no evidence of moderation by emotional eating tendencies. See Table 4.

In a set of additional analyses we examined whether there was any evidence for moderation of the effect of condition on intake for any of the other meal items or total energy intake. We found no evidence of moderation in these analyses (ps > 0.05).

## 4 Discussion

### 4.1 Primary findings

The primary aim of the present study was to examine whether heightening participants' awareness that their food intake was being measured during a multi-item laboratory meal affected food consumption. In line with recent findings [4], we found that the consumption of an energy dense snack food (cookies) was lower in a heightened awareness condition compared to a less aware condition; in our main analysis of the full sample this equated to a 26% reduction in cookie consumption and the size of this effect (*d* = 0.37) is comparable to that reported in a recently conducted meta-analysis examining the effect of heightened awareness on food intake; *d* = 0.45 [10]. The size of this effect was larger in sensitivity analyses in which participants were excluded based on the awareness manipulations not being effective. We found little evidence that consumption of any other high or low fat savoury or sweet items (sandwiches, sausage rolls, crisps, grapes, rice cakes) or total energy intake were substantially affected by the experimental condition that participants were assigned to. One interpretation of this finding is that only cookie consumption was reduced in the heightened awareness vs. less aware condition due to raised self-presentation concerns. Cookies are energy dense and high in both fat and sugar, so consuming a large amount of this specific food (as opposed to the other food items) may have raised particularly high concerns about appearing indulgent or greedy. However, specific testing of this proposition is required, because consumption of other ‘less healthy’ food items in the meal was not lower in the heightened awareness vs. less aware condition.

### 4.2 Secondary findings

The present study also made a novel contribution by examining whether the effect that heightened awareness of observation vs. less awareness of observation had on cookie intake was moderated by participant characteristics. The sample size in these analyses was limited (and in some cases *N* < 20 per individual cell) and a number of the effects observed were only marginally statistically significant, so caution is needed in interpretation of these exploratory analyses. Participant weight status and trait measures of both dietary restraint and disinhibited eating were found to moderate the effect of experimental condition on cookie consumption. Overweight females, but not normal weight females ate fewer grams of cookies under conditions of heightened awareness vs. less awareness. Individuals with low dietary restraint, but not high dietary restraint, and disinhibited eaters, but not non-disinhibited eaters, ate fewer grams of cookies under conditions of heightened awareness vs. less awareness. However, these findings do not all support our hypotheses regarding self-presentation concerns. In line with our hypotheses, it is feasible that overweight women may feel more conscious of their eating behaviour than normal weight women and so reduced their cookie consumption under conditions of heightened awareness, which is consistent with some findings examining omissions in dietary self-report [30], [31]. It is important to note that in the present study we found little evidence of heightened awareness of consumption reducing cookie consumption among normal weight participants, but in a previous study from our laboratory this was observed [4]. It may be the case that differences in study methodology can explain this effect. In the previous study participants were only provided with cookies to eat and this may have raised all participants' concerns over the appropriateness of the exact number of cookies to eat; whereas in the present study a variety of food items were provided which may have reduced concerns over the appropriateness of how many cookies to eat for normal weight participants. Moreover, it is generally assumed that individuals with high dietary restraint are more concerned about weight and conscious of their eating than individuals with low dietary restraint [23], [24], so we predicted that heightened awareness may be most likely to reduce food intake among highly restrained eaters. In the present study only participants with low dietary restraint ate less in a heightened awareness condition vs. the less aware condition.

A potential explanation of the present findings relates to how much these different participant sub-groups would normally eat (when awareness is not heightened). The instances in the present study in which participant sub-groups did not reduce their food consumption in the heightened awareness condition vs. the less aware condition may be due to these participants already having a relatively low consumption of cookies (as indicated by consumption data in the less aware condition). For example, the restrained eaters in the present study may have already been restraining their cookie consumption and therefore eating minimally, so the heightened awareness manipulation did not reduce cookie intake any further. Likewise, the same explanation may explain why both normal weight participants and low disinhibited eaters did not eat less under conditions of heightened awareness vs. less awareness. Regardless of why participant individual differences moderate the influence that heightened awareness of observation has on food intake, these findings may indicate that some participant sub-groups are more or less likely to alter their eating behaviour when feeling observed and this could in theory result in erroneous study conclusions if awareness of observation is not minimised. For example, if a specific participant sub-group in a study is eating minimally because of self-presentation concerns, this may produce a ‘floor effect’ which could diminish any resulting response to an experimentally manipulated variable.

### 4.3 Applied relevance

The validity and limitations of self-report forms of dietary assessment have been studied in some detail [11], [12], [14]. However, the direct effect that participant awareness of observation has on eating behaviour in the laboratory is a relatively new area of investigation. The present study builds on this research and therefore draws attention towards a lesser considered methodological issue in the study of human dietary behaviour. Laboratory methods are designed to understand human eating behaviour in a systematic and controlled fashion and ensuring that behaviour observed in the laboratory will translate to the real world is of obvious importance. To date, there has been little examination of how findings in the laboratory correspond to real world eating behaviour. Although some findings are replicated when studies are conducted in the laboratory and the field, e.g. the influence portion size has on energy intake [32], [33], other factors which have been shown to influence energy intake in the field are not always reliably replicated in the laboratory [34] and it remains to be seen whether a large number of factors which influence energy intake in the laboratory reliably influence energy intake in the field. One likely difference between laboratory and field studies is that in the field participants are less likely to realise food consumption or eating behaviour are the ‘focus’ of the research. For example, two meta-analyses have now suggested that the influence portion size and plate size have on food consumption may be reduced when participants are aware that they are participating in an eating study [32], [35]. Therefore, future work would benefit from formally assessing whether the environment in which an eating behaviour study is conducted affects the results observed. Here we focused exclusively on participants' awareness of their food consumption being measured during a study, although a different type of participant awareness is whether or not participants are aware of specific study hypotheses being tested; e.g. whether a manipulated independent variable affects the amount of food eaten. The degree to which participants are blinded to study hypotheses may be another issue of applied relevance which deserves attention, as often this information is not reported and if the hypotheses being tested are transparent, this may produce demand characteristics. Thus, understanding how we can design studies to ensure that findings from the laboratory translate to the real world is an important challenge and addressing participant awareness of observation may be one way of achieving this.

The extent to which the present findings have practical applications for the design of laboratory studies of eating behaviour requires consideration. Here we found little evidence that a number of test-meal items or total energy intake were affected by heightened awareness vs. less awareness, which could be interpreted as evidence that awareness of observation has little influence on energy intake of a multi-item test meal. However, it should be noted that in the present study we did not include an ‘unaware’ condition; namely a group of participants who did not believe (at all) that their food intake was being measured. Our less aware condition did indeed have a lower score on the awareness of observation manipulation check than the heightened awareness condition, but a relatively high percentage of participants in the less aware condition (approx. 67%, compared to 91% in the heightened awareness condition) did agree or strongly agree that they believed their food intake would be measured by the researcher. Thus, the present study is not well positioned to tell us whether *awareness* of observation affects consumption of different food items, but rather it tells us what effect *heightened awareness* has on eating behaviour. It is conceivable that if a study was particularly well disguised, the percentage of participants believing their food intake was being measured would be lower and this might result in participants eating more ‘naturally’ or ‘freely’. For example, rather than participating in a study framed as being related to food or eating (as was the case in the present study), food consumption could be presented to participants secondary to another activity unrelated to eating (e.g. [36]). It would seem likely that in the latter example participants would be less focused on their own eating behaviour. Thus, there are likely to be varying degrees of participant awareness in laboratory settings. These points considered, we suggest that minimizing participant awareness of observation during laboratory eating behaviour studies may be necessary and the present study results suggest that this is likely to be of particular importance when studies use foods which may invoke self-presentation concerns among females. As discussed elsewhere [4], [10], formally measuring awareness of observation and participant beliefs about study aims may also aid researchers in interpreting the robustness of their findings.

### 4.4 Limitations

The present study sampled females only so we are unable to draw any conclusions about how heightened awareness of observation affects food intake in males. It may be the case that the individual differences identified in the present study also apply to male populations, but further research will need to directly address this. We also examined food consumption of a small number of food items (which were all non-amorphous) in a single session study. Whether other foods would be affected by awareness of observation (e.g. amorphous foods, such as soup or pasta) is therefore not clear. Likewise, the extent to which heightened awareness of observation may affect energy intake when participants make multiple visits to the laboratory remains unknown. It could be argued that self-presentation concerns would reduce over time and if this were the case then the use of repeated measures/within subjects designs could be a way of minimizing the effects of heightened awareness of observation. Yet, an issue with the use of repeated measures/within subject designs is that participants may be more likely to become aware of the hypotheses being tested and alter their eating behaviour accordingly. As noted, we had relatively small samples sizes when examining the effect of heightened awareness in moderation analyses and the results of these analyses should be interpreted with caution. Further direct testing of potential moderators of the influence that heightened awareness has on food intake in the laboratory is therefore needed before strong conclusions can be drawn. Finally, as discussed, a relatively large proportion of participants in our ‘less aware’ condition reported awareness of their food consumption being measured. A further ‘control’ condition in which participant awareness of observation is very low would be useful in future research in order to increase confidence that differences in awareness of observation are responsible for between-condition differences in food intake.

## 5 Conclusions

Heightened awareness of observation may cause females to reduce their consumption of an energy dense snack food during a test meal in the laboratory and this effect may be moderated by participant individual differences. These results further suggest that minimizing participant awareness of observation during laboratory eating behaviour studies is of importance.

## Authorship

ER and UM conceived the study. MO and MP oversaw data collection. ER and MO analysed study data. ER drafted the manuscript. All authors approved the final manuscript.

## Conflicts of interest

All authors report there are no conflicts of interest relating to this work.

## Funding

ER was supported by the Wellcome Trust, grant number 097826/Z/11/A.

## Figures and Tables

**Table 1:** Items provided at the ad libitum lunch per serving (g) (LFSA — Low Fat Savoury, HFSA — High Fat Savoury, LFSW — Low Fat Sweet, HFSW — High Fat Sweet).

Item	Serving(g)	k cal/serving	Fat/serving	CHO/serving	PRO/serving
Ham sandwich (LFSA)	165	339.9	4.95	48.5	20.3
Mini sausage rolls (HFSA)	102	316.2	17.4	29.4	9.0
Salt and vinegar crisps (HFSA)	50	259.5	15.4	26.4	3.0
Salt and vinegar rice cakes (LFSA)	44	178.6	3.2	34.0	3.0
Chocolate chip cookies (HFSW)	104	506.5	23.5	66.4	5.6
Green seedless grapes (LFSW)	230	151.8	0.2	35.4	0.9

**Table 2:** Sample characteristics.

	Less Aware, *N* = 60	Heightened Awareness *N* = 54	*t*-test results
Age (years)^a^	23.47 (8.53)	25.15 (12.41)	t (111) = − 0.85, *p* = 0.40, *d* = 0.16
BMI (kg/m^2^)^b^	23.68 (4.85)	23.53 (4.35)	t (109) = 0.17, *p* = 0.87, *d* = 0.03
Baseline hunger (0–100 mm line scale)	6.64 (1.76)	6.56 (1.58)	t (112) = 0.27, *p* = 0.79, *d* = 0.05
Restraint (1–4 score)	2.31 (0.62)	2.47 (0.65)	t (112) = − 1.32, *p* = 0.19, *d* = 0.25
Disinhibition (1–4 score)	2.44 (0.52)	2.50 (0.52)	t (112) = − 0.69, *p* = 0.49, *d* = 0.12
Emotional eating (1–4 score)	2.24(0.70)	2.33 (0.78)	t (112) = − 0.64, *p* = 0.53, *d* = 0.12

Values refer to means, brackets denote standard deviation. Dietary restraint, disinhibition and emotional eating are represented by a 1–4 score, with higher scores denoting higher dietary restraint, dietary disinhibition and emotional eating, *t*-test results refer to mean difference between less aware condition and heightened awareness condition.

a One participant opted not to report their age.

b Measurement error resulted in missing BMI data for three participants.

**Table 3:** Consumption of test-meal foods.

	Less aware, *N* = 60	Heightened awareness *N* = 54	*t*-Test results
Cookies (grams)	25.33 (19.47)	18.75 (15.67)	t (112) = 1.98, *p* = 0.05, *d* = 0.37
Sausage rolls (grams)	32.55 (26.88)	29.53 (27.44)	t (112) = 0.59, *p* = 0.55, *d* = 0.11
Crisps (grams)	12.65 (12.38)	14.27(12.69)	t (112) = − 0.69, *p* = 0.49, *d* = 0.13
Grapes (grams)	99.35 (59.46)	106.27(63.67)	t (112) = − 0.60, *p* = 0.55, *d* = 0.11
Sandwiches (grams)	87.20 (46.48)	84.19 (40.74)	t (112) = 0.37, *p* = 0.72, *d* = 0.07
Rice cakes (grams)	10.82 (12.84)	13.53 (13.53)	t (112) = − 1.10, *p* = 0.28, *d* = 0.21
Total energy intake (k cals)	579.05 (185.62)	555.40 (176.33)	t (112) = 0.70, *p* = 0.49, *d* = 0.13

Values refer to mean grams of food item consumed, brackets denote standard deviation, *t*-test results refer to mean difference between less aware condition and heightened awareness condition.

**Table 4:** Results of moderation analyses for cookie intake.

		Less aware	Heightened awareness	*t*-test results
Weight status	Normal weight^a^	22.64 (17.99), *N* = 37	22 (17.26), *N* = 34	t (69) = 0.15, *p* = 0.88, *d* = 0.04
Overweight/obese^a^	29.36 (19.94). *N* = 16	12.47 (10.96), *N* = 16	t (30) = 2.97, *p* = 0.01, *d* = 1.05
Restraint	Low restraint	30.85 (21.30), *N* = 35	19.47 (15.81), *N* = 22	t (55) = 2.16, *p* = 0.04, *d* = 0.61
High restraint	17.59 (13.49), *N* = 25	18.25 (15.80), *N* = 32	t (55) = − 0.17, *p* = 0.87, *d* = 0.04
Disinhibition	Low disinhibition	19.58 (17.08), *N* = 34	17.26 (14.86), *N* = 30	t (62) = 0.58, *p* = 0.57, *d* = 0.14
High disinhibition	32.85 (20.13), *N* = 26	20.61 (16.75), *N* = 24	t (48) = 2.33, *p* = 0.02, *d* = 0.66
Emotional eating	Low emotional eating	24.46 (20.96), *N* = 28	18.82 (15.08), *N* = 30	N/A — lack of interaction effect in ANCOVA
High emotional eating	26.09 (18.36), *N* = 32	18.65 (16.70), *N* = 24	

Values refer to mean grams of cookies consumed, brackets denote standard deviation, N = number of participants, t-test results refer to mean difference between less aware condition and heightened awareness condition for cookie consumption.

a Normal weight M BMI = 21.8, Overweight/Obese M BMI = 29.1.

## References

[1] Meiselman H.L. (1992). Methodology and theory in human eating research. Appetite.

[2] de Castro J.M. (2000). Eating behavior: lessons from the real world of humans. Nutrition.

[3] Blundell J., De Graaf C., Hulshof T. (2010). Appetite control: methodological aspects of the evaluation of foods. Obes. Rev..

[4] Robinson E., Kersbergen I., Brunstrom J.M. (2014). I'm watching you. Awareness that food consumption is being monitored is a demand characteristic in eating-behaviour experiments. Appetite.

[5] Oakes M.E. (2004). Slotterback CS Prejudgments of those who eat a “healthy” versus an “unhealthy” food for breakfast. Curr. Psychol..

[6] Pliner P., Chaiken S. (1990). Eating, social motives, and self-presentation in women and men. J. Exp. Soc. Psychol..

[7] Mori D., Chaiken S., Pliner P. (1987). “Eating lightly” and the self-presentation of femininity. J. Pers. Soc. Psychol..

[8] Bock B.C., Kanarek R.B. (1995). Women and men are what they eat: the effects of gender and reported meal size on perceived characteristics. Sex Roles.

[9] Roth D.A., Herman C.P., Polivy J. (2001). Self-presentational conflict in social eating situations: a normative perspective. Appetite.

[10] Robinson E., Hardman C.A., Halford J.C. (2015). Eating under observation: a systematic review and meta-analysis of the effect that heightened awareness of observation has on laboratory measured energy intake. Am. J. Clin. Nutr..

[11] Gemming L., Ni Mhurchu C. (2016). Dietary under-reporting: what foods and which meals are typically under-reported?. Eur. J. Clin. Nutr..

[12] Lafay L., Mennen L., Basdevant A. (2000). Does energy intake underreporting involve all kinds of food or only specific food items? Results from the Fleurbaix Laventie Ville Sante (FLVS) study. Int. J. Obes..

[13] Macdiarmid J., Blundell J. (1998). Assessing dietary intake: who, what and why of under-reporting. Nutr. Res. Rev..

[14] Stubbs R.J., O'Reilly L.M., Whybrow S. (2014). Measuring the difference between actual and reported food intakes in the context of energy balance under laboratory conditions. Br. J. Nutr..

[15] Thomas J.M., Dourish C.T., Higgs S. (2015). Effects of awareness that food intake is being measured by a universal eating monitor on the consumption of a pasta lunch and a cookie snack in healthy female volunteers. Appetite.

[16] Robinson E., Higgs S. (2013). Food choices in the presence of ‘healthy’ and ‘unhealthy’ eating partners. Br. J. Nutr..

[17] Haynes A., Kemps E., Moffitt R. (2015). The moderating role of state inhibitory control in the effect of evaluative conditioning on temptation and unhealthy snacking. Physiol. Behav..

[18] Rolls B.J., Roe L.S., Halverson K.H. (2007). Using a smaller plate did not reduce energy intake at meals. Appetite.

[19] Péneau S., Mekhmoukh A., Chapelot D. (2009). Influence of environmental factors on food intake and choice of beverage during meals in teenagers: a laboratory study. Br. J. Nutr..

[20] Cappelleri J.C., Bushmakin A.G., Gerber R.A. (2009). Psychometric analysis of the three-factor eating questionnaire-R21: results from a large diverse sample of obese and non-obese participants. Int. J. Obes..

[21] Lee A.B., Goldman M. (1979). Effect of staring on normal and overweight students. J. Soc. Psychol..

[22] Major B., Eliezer D., Rieck H. (2012). The psychological weight of weight stigma. Soc. Psychol. Personal. Sci..

[23] Polivy J., Herman C.P., Hackett R. (1986). The effects of self-attention and public attention on eating in restrained and unrestrained subjects. J. Pers. Soc. Psychol..

[24] Rennie K.L., Siervo M., Jebb S.A. (2006). Can self-reported dieting and dietary restraint identify underreporters of energy intake in dietary surveys?. J. Am. Diet. Assoc..

[25] Bryant E.J., King N.A., Blundell J.E. (2008). Disinhibition: its effects on appetite and weight regulation. Obes. Rev..

[26] van Strien T., Cebolla A., Etchemendy E. (2013). Emotional eating and food intake after sadness and joy. Appetite.

[27] Oliver G., Wardle J., Gibson E.L. (2000). Stress and food choice: a laboratory study. Psychosom. Med..

[28] Robinson E., Blissett J., Higgs S. (2011). Peak and end effects on remembered enjoyment of eating in low and high restrained eaters. Appetite.

[29] Masic U., Yeomans M.R. (2014). Umami flavor enhances appetite but also increases satiety. Am. J. Clin. Nutr..

[30] Heitmann B.L., Lissner L. (1995). Dietary underreporting by obese individuals—is it specific or non-specific?. BMJ.

[31] Schoeller D.A., Thomas D., Archer E. (2013). Self-report-based estimates of energy intake offer an inadequate basis for scientific conclusions. Am. J. Clin. Nutr..

[32] Zlatevska N., Dubelaar C., Holden S.S. (2014). Sizing up the effect of portion size on consumption: a meta-analytic review. J. Mark..

[33] Robinson E., te Raa W., Hardman C.A. (2015). Portion size and intended consumption. Evidence for a pre-consumption portion size effect in males?. Appetite.

[34] Robinson E., Nolan S., Tudur-Smith C. (2014). Will smaller plates lead to smaller waists? A systematic review and meta-analysis of the effect that experimental manipulation of dishware size has on energy consumption. Obes. Rev..

[35] Holden S., Zlatevska N., Dubelaar C. (2015). Whether smaller plates reduce consumption depends on who's serving and who's looking: a meta-analysis. J. Assoc. Consum. Res..

[36] Hermans R.C., Larsen J.K., Herman C.P., Engels R.C. (2009). Effects of social modeling on young women's nutrient-dense food intake. Appetite.

